# Comparative mitochondrial genomics of cultivated *Sesamum indicum* and its wild relative *Sesamum schinzianum* provides insights into structural features and organellar evolution

**DOI:** 10.3389/fpls.2026.1858359

**Published:** 2026-06-10

**Authors:** Junchao Liang, Xiaowen Yan, Zhiqi Wang, Pan Zeng, Shixiang Li, Jian Sun

**Affiliations:** Crop Research Institute, Jiangxi Academy of Agricultural Sciences, Jiangxi Province Key Laboratory for Genetic Improvement of Oil Crops, Nanchang, China

**Keywords:** comparative genomics, genomic evolution, mitochondrial genome, phylogenetics, sesame

## Abstract

**Introduction:**

The genus *Sesamum* (Pedaliaceae) comprises a wide range of cultivated and wild species. Sesame (*Sesamum indicum*) is recognized as one of the oldest oilseed crops cultivated worldwide, whereas *S. schinzianum* is a wild relative closely associated with the evolutionary history of cultivated sesame. Although the nuclear and chloroplast genomes of *Sesamum* species have been investigated in previous studies, mitochondrial genome evolution within the genus has received relatively limited attention.

**Methods:**

In this study, we assembled and comparatively analyzed the mitochondrial genomes of *S. indicum* and *S. schinzianum* by integrating BGI short-read sequencing and Oxford Nanopore long-read sequencing data. Genome assembly was performed using Flye and Unicycler, and annotation was conducted using PMGA together with manual curation. Comparative analyses were then carried out to examine genome organization, gene content, repetitive sequences, codon usage, RNA editing sites, chloroplast-derived sequences, phylogenetic relationships, and collinearity patterns.

**Results:**

The mitochondrial genomes of *S. indicum* and *S. schinzianum* were assembled into one circular molecule and two major circular contigs, respectively, and both contained 36 conserved protein-coding genes. Abundant simple sequence repeats dominated by tetranucleotide motifs and notable repeat variation were detected. Codon usage showed moderate bias, and 478 and 455 RNA editing sites were predicted in *S. indicum* and *S. schinzianum*, respectively. Chloroplast-derived sequences accounted for 8.50% and 6.96% of the mitochondrial genomes, respectively. Phylogenetic and collinearity analyses supported a close relationship between the two *Sesamum* species and identified synteny-based structural differences between their mitogenomes. Comparative analysis of mitochondrial- and chloroplast-based phylogenies showed that the two datasets were largely congruent at the family level and consistently supported the close relationship between the two Sesamum species, although they differed in the placement of several deeper lineages.

**Discussion:**

These results suggest that *Sesamum* mitogenomes retain conserved gene content while showing assembly- and synteny-supported structural differences. This study provides useful genomic resources for comparative and evolutionary studies of mitochondrial genomes in Pedaliaceae.

## 1 Introduction

*Sesamum* L. is the largest genus within the family Pedaliaceae, comprising approximately 31 species (POWO, 2026). Species of this genus are naturally distributed across tropical and subtropical regions of Africa, South and Southeast Asia, Melanesia, and Australia (Gormley et al., 2015; POWO, 2026). Among them, *S. indicum* is widely cultivated in Africa and Asia for its high-quality seeds and is recognized as one of the oldest oilseed crops, often referred to as the “queen of oilseeds” (Angamuthu et al., 2025). With the continuous growth of the global population, the demand for high-quality edible oils is steadily increasing. Sesame oil is rich in polyunsaturated fatty acids, which are associated with significant health benefits, making it an important oilseed resource (Mehra, 1970).

From an evolutionary perspective, cultivated sesame and its wild relatives provide an informative system for investigating organellar genome evolution during species divergence and crop domestication. Plant mitochondrial genomes are characterized by conserved coding sequences but highly variable structures, and comparisons between cultivated and wild taxa can help reveal lineage-specific rearrangements, repeat dynamics, and intracellular DNA transfer events. From an identification perspective, mitochondrial genome features, including repeat profiles, species-specific transferred fragments, and structural variations, may provide useful molecular information for distinguishing closely related *Sesamum* germplasm. From a breeding perspective, wild *Sesamum* species represent valuable genetic resources for improving stress tolerance, environmental adaptability, and cytoplasmic diversity in sesame breeding programs. Therefore, mitochondrial genomic comparisons between cultivated sesame and wild relatives are important not only for understanding organellar genome evolution but also for supporting germplasm identification and future genetic improvement.

The wild tetraploid species *S. schinzianum* is considered a potential ancestor of cultivated diploid sesame and is native to tropical desert regions of the African plateau (Wang et al., 2023; POWO, 2026). In these environments, plants are exposed to extreme abiotic stresses, including high temperatures, drought, and intense radiation, which impose substantial metabolic and energetic challenges (Qiao et al., 2016).

Mitochondria, as the primary sites of energy metabolism, function as the “powerhouses” of eukaryotic cells by generating ATP through cellular respiration (Saraste, 1999; Roger et al., 2017). Plant mitochondrial genomes exhibit remarkable size variation, spanning approximately 66 kb to 11.3 Mb (Sloan et al., 2012; Skippington et al., 2015), a pattern that is largely driven by differences in non-coding DNA. For example, in Fagaceae, dispersed repeats have been identified as a major factor influencing mitochondrial genome size (Tu et al., 2024), whereas in *Cucurbita*, the nearly 1 Mb mitochondrial genome is primarily attributed to the accumulation of foreign chloroplast-derived sequences and the expansion of small repeats (Alverson et al., 2010).

Although mitochondrial genomes in plants are often represented as circular structures, they can also exist in linear, branched, or multipartite circular forms (Sloan, 2013; Kozik et al., 2019). Such structural diversity may enhance the flexibility of gene expression and thereby facilitate plant adaptation to environmental conditions. In addition, these genomes often experience repeat-mediated homologous recombination, resulting in the formation of subgenomic molecules or structural isoforms (Cole et al., 2018; Odahara et al., 2021).

Due to the abundance of repetitive sequences and extensive genomic recombination, resolving the true configuration of plant mitochondrial DNA (mtDNA) remains challenging. However, with the rapid development of third-generation sequencing technologies and improved assembly strategies, it is becoming increasingly feasible to uncover the complex architectures of plant mitochondrial genomes.

Among the approximately 31 recognized *Sesamum* species, *S. indicum* and *S. schinzianum* were selected because they represent two biologically and evolutionarily important lineages within the genus *S. indicum* is the only widely domesticated and globally cultivated sesame species, and therefore represents the cultivated gene pool of *Sesamum*. In contrast, *S. schinzianum* is a wild relative and putative progenitor-related species that occurs in arid and semi-arid environments and has been considered important for understanding the origin, adaptation, and diversification of cultivated sesame. Previous studies have attempted distant hybridization between *S. indicum* and *S. schinzianum* to introduce beneficial resistance-related traits from the wild species into cultivated sesame (Zhang et al., 2013). Therefore, *S. schinzianum* is valuable not only for understanding the evolutionary history and diversification of *Sesamum*, but also for providing potential genetic resources for sesame improvement. The comparison between these two species thus provides a suitable cultivated–wild framework for evaluating mitochondrial genome conservation and divergence in *Sesamum*. In addition, a mitochondrial genome of *S. indicum* has recently been reported by Wang et al. (2024), providing an important reference for assessing intraspecific structural conservation, assembly differences, and lineage-specific variation. By comparing our newly assembled *S. indicum* mitogenome with the published *S. indicum* genome and the wild relative *S. schinzianum*, we aimed to clarify both intraspecific and interspecific patterns of mitochondrial genome evolution in *Sesamum*.

Recent advances in long-read sequencing have greatly improved the assembly of plant mitochondrial genomes, especially in resolving repeat-rich and structurally complex regions. Short-read sequencing alone is often insufficient to distinguish long repeats and alternative configurations, whereas long-read sequencing provides greater continuity for resolving complex assembly graphs. Hybrid strategies that combine short reads and long reads can further improve base-level accuracy while retaining long-range structural information. For *Sesamum*, Wang et al. (2024) recently reported the first complete mitochondrial genome of *S. indicum* based on third-generation sequencing data, providing an important reference for mitochondrial genome research in this genus. However, mitochondrial genomic information remains limited for wild *Sesamum* relatives, and comparative analyses between cultivated sesame and wild relatives are still insufficient. In addition, independent assemblies of cultivated *S. indicum* based on different sequencing platforms and analytical pipelines can help evaluate intraspecific conservation, assembly consistency, and potential lineage-specific structural features.

In this work, we generated high-quality mitochondrial genome assemblies for *S. indicum* and *S. schinzianum* by integrating BGI short-read data with Nanopore long-read data. We conducted detailed analyses of gene content, codon usage bias, RNA editing sites, repeat sequences, genome rearrangements, intracellular sequence transfer, and phylogenetic relationships. By characterizing the mitochondrial genomes of cultivated sesame and its wild counterpart *S. schinzianum*, our study offers valuable insights into the evolutionary patterns of plant mitochondrial genomes and enhances understanding of plant adaptation to extreme environments. Compared with previous studies focusing primarily on cultivated *S. indicum*, this study integrates newly assembled mitogenomes of cultivated *S. indicum* and the wild relative *S. schinzianum*, together with the published *S. indicum* mitogenome, to provide a cultivated–wild comparative framework for assessing gene content conservation, repeat composition, plastid-to-mitochondrion DNA transfer, synteny-based structural features, and organellar phylogenetic relationships in *Sesamum*.

## 2 Materials and methods

### 2.1 Plant material, DNA extraction, and sequencing

Plant materials of *Sesamum indicum* cultivar ‘Zhongzhi No.11’ (**Supplementary Figure 1**) and wild *S. schinzianum* (‘Gangguo yezhima’) (**Supplementary Figure 2**) were obtained from the sesame germplasm repository at the Nanchang Branch of the National Center for Oil Crops Improvement, Jiangxi Academy of Agricultural Sciences, China. Species identification was performed by Jian Sun. Voucher specimens of *S. indicum* and *S. schinzianum* were deposited in the sesame germplasm repository of the Crop Research Institute, Jiangxi Academy of Agricultural Sciences, No. 602 Nanlian Road, Nanchang, Jiangxi, China, under voucher numbers ZZ11 and GY, respectively. Total DNA was extracted from fresh leaves using a modified CTAB method as described by Doyle (Doyle and Doyle, 1987). Sequencing was conducted using the BGI sequencing platform (BGI, Shenzhen, China) and the Oxford Nanopore platform (Oxford Nanopore Technologies, Oxford, UK).

### 2.2 Assembly and annotation

To obtain high-quality clean reads, raw short reads were filtered using Trimmomatic v0.40 (Bolger et al., 2014), while raw long reads were corrected and trimmed using Canu v2.3 (Koren et al., 2017), respectively. Long-read sequencing data were first assembled using Flye v2.9.6 (Kolmogorov et al., 2019) with default parameters, generating a graph-based assembly in GFA format. All contigs in FASTA format were used to build a local database, and candidate mitochondrial contigs were identified by BLASTN v2.13.0 (Chen et al., 2015) searches against an expanded local mitochondrial reference database. This database was constructed by integrating the plant mitochondrial core gene database (https://github.com/xul962464/plant_mt_ref_gene) with available *Sesamum* mitochondrial genome resources, including the previously published *S. indicum* mitogenome (GenBank accession PP444661-PP444682). The BLASTN parameters were meter”-evalue 1e-5 -outfmt 6 -max_hsps 10 -word_size 7 -task blastn-short”. Candidate mitochondrial contigs were further evaluated based on mitochondrial gene hits, assembly graph topology, and long-read mapping support. The assembly graph was visualized using Bandage v0.8.1 (Wick et al., 2015). Long reads were then mapped to the mitochondrial contigs using BWA v0.7.17 (Li, 2013), and the mapped reads were extracted to resolve repeat regions in the mitochondrial assembly graph. Subsequently, short reads generated from the BGI platform were also aligned to the mitochondrial contigs using BWA v0.7.17 (Li, 2013), and the corresponding reads were extracted. Finally, hybrid assembly was performed using Unicycler v0.5.1 (Wick et al., 2017) based on the extracted mitochondrial long and short reads to obtain the complete mitochondrial genome sequences of *S. indicum* and *S. schinzianum*.

The mitochondrial genome was annotated using the online platform PMGA (Li et al., 2025), and the annotations were further manually curated in Geneious v11.1.5 (Kearse et al., 2012). The mitochondrial genome map was visualized using OGDRAW v1.3.1 (Greiner et al., 2019).

### 2.3 Repeat sequences analysis and Ka/Ks variation

SSRs in the mitochondrial genome were identified using the MISA web server (Beier et al., 2017) with minimum repeat thresholds of 10 for mononucleotides, 5 for dinucleotides, 4 for trinucleotides, and 3 for tetranucleotide, pentanucleotides, and hexanucleotide motifs. Tandem repeat sequences were subsequently examined using the Finder web service (Benson, 1999) under default settings. Dispersed repeats were detected with the REPuter web server (Kurtz et al., 2001), applying a Hamming distance of 3, a minimum repeat length of 30 bp, and a maximum repeat number of 5000.

KaKs_Calculator v2.0 (Wang et al., 2010) was used to calculate the nonsynonymous to synonymous substitution rates (Ka/Ks) ratios of all protein-coding genes (PCGs) in the *Sesamum* mitochondrial genome. This ratio reflects the relative rates of nonsynonymous and synonymous substitutions.

### 2.4 Codon usage bias and RNA editing sites analysis

Protein-coding genes were extracted from the mitochondrial genomes using PhyloSuite v.1.1.16 (Zhang et al., 2020). Codon usage and RSCU values were calculated for unique PCGs using CodonW (https://codonw.sourceforge.net/).

RNA editing sites in the mitochondrial genome were identified using Deepred-Mt (Edera et al., 2021) with a prediction probability threshold of ≥ 0.9.

### 2.5 Intracellular gene transfer and collinearity analysis

The chloroplast genomes of *S. indicum* and *S. schinzianum* were assembled from the same BGI short-read datasets generated for mitochondrial genome assembly. Although chloroplast genomes of *Sesamum* species have been reported previously, we reassembled the chloroplast genomes from the same plant materials used for mitogenome assembly to ensure sample consistency in the identification of plastid-derived mitochondrial sequences. GetOrganelle v1.7.1 (Jin et al., 2020) was used for chloroplast genome assembly, and PGA (Qu et al., 2019) was used for annotation. Homologous regions between the mitochondrial genome and the chloroplast genome were identified through sequence similarity searches using BLASTN v2.13.0 (Chen et al., 2015) with an E-value cutoff of 1e-5. The resulting alignments were subsequently visualized using the online platform circoletto (Darzentas, 2010).

Multiple sequence alignment of mitochondrial genomes from species within the genus *Sesamum* was performed using AliTV v1.0.6 (Ankenbrand et al., 2017). To improve the clarity of the visualization, only alignments with sequence similarity greater than 70% and lengths exceeding 200 bp were displayed.

### 2.6 Phylogenetic analysis

Phylogenetic relationships among species were inferred by constructing a phylogenetic tree using conserved mitochondrial PCGs. Conserved mitochondrial PCGs were extracted from the genomes using PhyloSuite v1.1.16 (Zhang et al., 2020) for subsequent phylogenetic analysis. For phylogenetic reconstruction, mitochondrial PCGs were extracted from all sampled Lamiales mitogenomes using PhyloSuite v1.1.16. The 36 non-redundant PCGs reported for the newly assembled *Sesamum* mitogenomes refer specifically to the gene content of the two *Sesamum* species. For the broader Lamiales-wide phylogenetic dataset, 38 mitochondrial PCG loci were initially retained because two additional genes, *rpl*23 and *rps*7, were present in most sampled Lamiales species and provided additional phylogenetic information. Duplicated, incomplete, and ambiguously annotated sequences were removed. For genes with multiple annotated copies or fragmented annotations, only one representative orthologous sequence was retained based on sequence completeness and annotation consistency. Genes absent from a small number of taxa were treated as missing data in the concatenated alignment. The 38 PCG sequences were aligned using MAFFT v7.490 (Katoh and Standley, 2013) with default parameters, and poorly aligned regions were automatically removed using trimAl v1.2 (Capella-Gutiérrez et al., 2009) to retain high-quality alignment sites. The filtered PCG alignments were concatenated into a single supermatrix, which was then used for phylogenetic reconstruction with IQ-TREE v2.0.3 (Minh et al., 2020). Branch support was assessed using the SH-aLRT test and ultrafast bootstrap (UFBoot) with 1,000 replicates each, and the best-fit substitution model was automatically selected by ModelFinder. Phylogenetic analyses based on plastid DNA were performed using the same pipeline as those based on mitochondrial DNA. The GenBank accession numbers of the mitochondrial genome and plastid genome used for constructing the phylogenetic tree in this study are shown in **Supplementary Table 1**.

## 3 Results

### 3.1 Features of the mitogenomes

**Figure 1:**
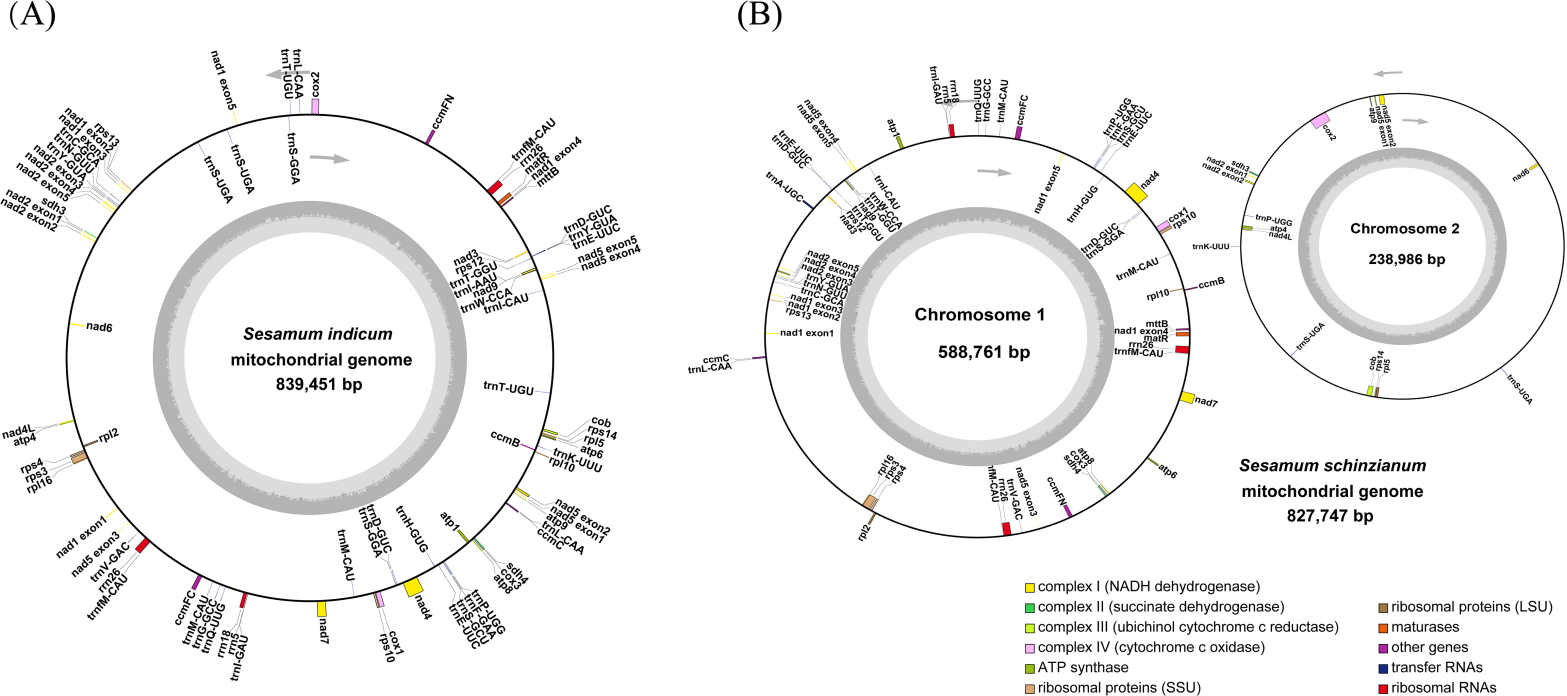
The complete mitochondrial genome map of *S. indicum* **(A)** and *S. schinzianum* **(B)**. Genes belonging to different functional groups are color-coded.

For *S. indicum*, 9.99 Gb of raw Nanopore sequencing data and 11.26 Gb of raw BGI sequencing data were generated. For *S. schinzianum*, 16.61 Gb of raw Nanopore data and 11.02 Gb of raw BGI data were produced. Based on the assembly graph and long-read-supported contig connections, the mitochondrial genome assembly of *S. indicum* was represented as one major circular molecule of 839,451 bp, with an overall GC content of 44.28% (**Figure 1A**). After resolving repeat-mediated regions using Nanopore long reads, one predominant circular contig was obtained. The mitochondrial genome assembly of *S. schinzianum* showed a more complex assembly graph and was represented by two major circular contigs with a combined length of 827,747 bp and a GC content of 44.35% (**Figure 1B**). Because plant mitochondrial genomes may exist as dynamic and alternative molecular configurations *in vivo*, these circular representations should be interpreted as assembly-based structural models rather than direct evidence of the predominant physical form in cells.

Annotation of the two mitogenomes identified the same set of 36 distinct protein-coding gene types in both species (**Table 1**). In *S. schinzianum*, *nad*2 and *nad*5 were annotated at duplicated loci with different intron structures. These duplicated annotations were therefore treated as duplicated annotated loci rather than additional distinct PCG types. These included five ATP synthase genes (*atp*1, *atp*4, *atp*6, *atp*8, and *atp*9), four cytochrome c biogenesis genes (*ccmB, ccmC, ccmFC*, and *ccmFN*), three cytochrome c oxidase genes (*cox*1, *cox*2, and *cox*3), one ubiquinol cytochrome c reductase gene (*cob*), one maturase gene (*matR*), one transport membrane protein gene (*mttB*), nine NADH dehydrogenase genes (*nad*1, *nad*2, *nad*3, *nad*4, *nad*4L, *nad*5, *nad*6, *nad*7, and *nad*9), ten ribosomal protein genes, and two succinate dehydrogenase genes (*sdh*3 and *sdh*4).

**Table 1:** Mitochondrial genome gene composition of *S. indicum* and *S. schinzianum*.

Group of genes	Gene name of *S. indicum*	Gene name of *S. schinzianum*
ATP synthase	*atp*1, *atp*4, *atp*6, *atp*8, *atp*9	*atp*1, *atp*4, *atp*6, *atp*8, *atp*9
Cytochrome c biogenesis	ccmB, ccmC, ccmFC*, ccmFN*	ccmB, ccmC, ccmFC*, ccmFN
Ubiquinol cytochrome c reductase	*cob*	*cob*
Cytochrome c oxidase	*cox*1*, cox2*, *cox*3	*cox*1*, cox2*, *cox*3
Maturases	*matR*	*matR*
Transport membrane protein	*mttB*	*mttB*
NADH dehydrogenase	*nad*1****, nad2****, *nad*3, *nad*4***, *nad*4L, *nad*5****, *nad*6, *nad*7***, *nad*9	*nad*1****, *nad*2*, *nad*2_dup**, *nad*3, *nad*4***, *nad*4*L, nad*5*, *nad*5_dup**, *nad*6, *nad*7***, *nad*9
Ribosomal proteins (LSU)	*rpl*10, *rpl*16, *rpl*2, *rpl*5	*rpl*10, *rpl*16, *rpl*2, *rpl*5
Ribosomal proteins (SSU)	*rps*10*, rps12, *rps*13, *rps*14, *rps*3*, rps4	*rps*10*, rps12, *rps*13, *rps*14, *rps*3*, rps4
Succinate dehydrogenase	*sdh*3, *sdh*4	*sdh*3, *sdh*4
Ribosomal RNAs	*rrn*18, *rrn*26(2), *rrn*5	*rrn*18, *rrn*26(2), *rrn*5
Transfer RNAs	*trnC-GCA, trnD-GUC*(2), *trnE-UUC*(2), trnF-GAA, trnG-GCC, trnH-GUG, trnI-AAU*, trnI-CAU, trnI-GAU, trnK-UUU, trnL-CAA(2), *trnM-CAU*(2), *trnN-GUU, trnP-UGG, trnQ-UUG, trnS-GCU, trnS-GGA*(2), *trnS-UGA*(2), *trnT-GGU, trnT-UGU*(2), *trnV-GAC, trnW-CCA, trnY-GUA*(2), *trnfM-CAU*(2)	trnA-UGC*, trnC-GCA, trnD-GUC(2), *trnE-UUC*(2), *trnF-GAA, trnG-GCC, trnH-GUG, trnI-CAU, trnI-GAU, trnK-UUU, trnL-CAA, trnM-CAU*(2), *trnN-GUU, trnP-UGG*(2), *trnQ-UUG, trnS-GCU, trnS-GGA, trnS-UGA*(2), *trnT-GGU*(2), *trnV-GAC, trnW-CCA, trnY-GUA, trnfM-CAU*(2)

*intron number; Gene(2): Number of copies of multi-copy genes.

In *S. indicum*, 33 tRNA gene copies representing 24 unique tRNA loci and three rRNA genes (*rrn*5, *rrn*18, and *rrn*26, with *rrn*26 duplicated) were annotated. In *S. schinzianum*, 30 tRNA gene copies representing 23 unique tRNA loci and the same three rRNA genes were identified, with *rrn*26 also present in two copies. Compared with *S. indicum*, the *S. schinzianum* mitogenome uniquely contained *trnA-UGC*, whereas differences were also observed in the presence or copy number of several other tRNA genes.

### 3.2 Repetitive sequences and selection pressure in mitogenomes

**Figure 2:**
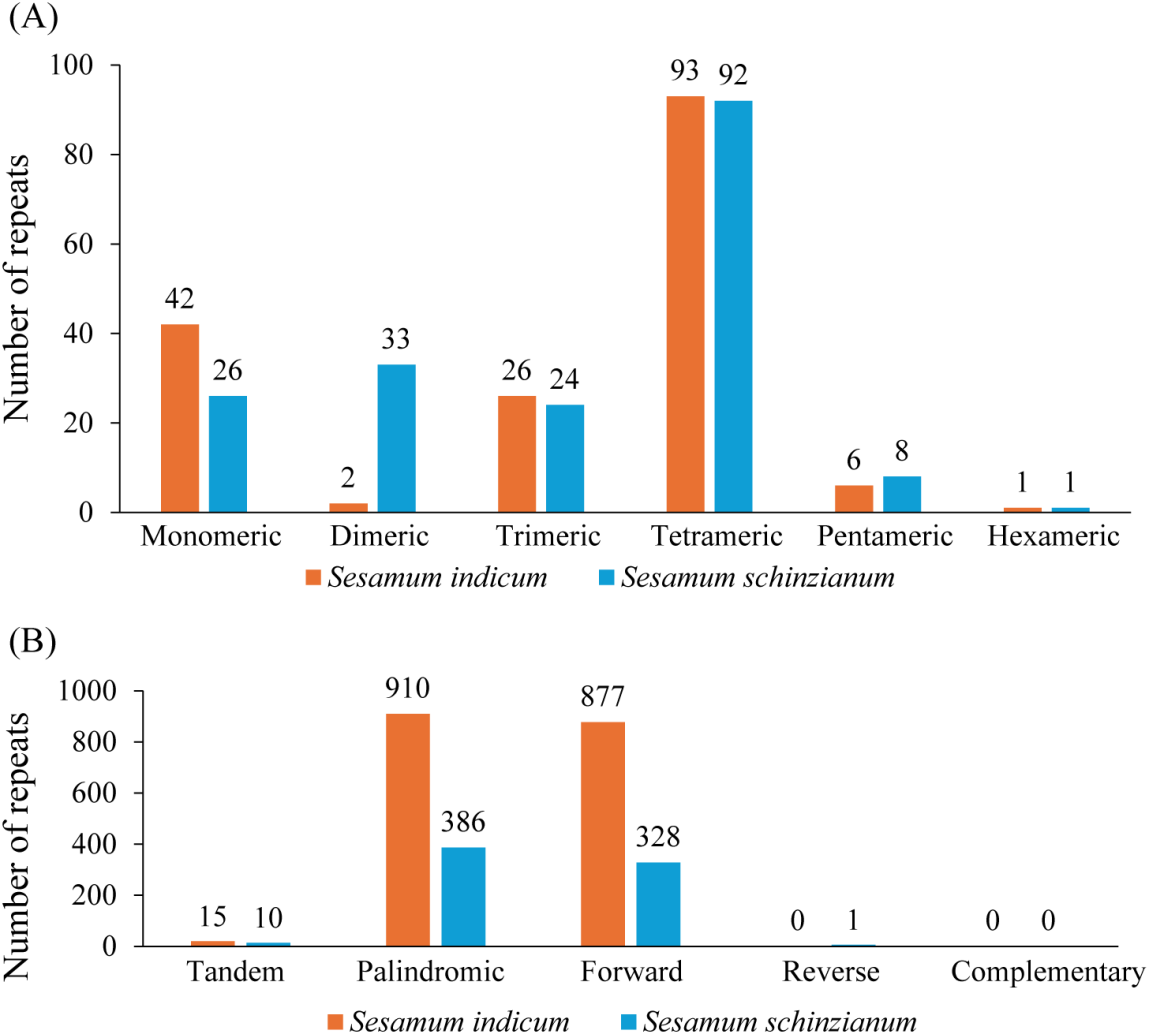
Characterization of simple sequence repeats (SSRs) and repeat elements in mitochondrial genomes of *S. indicum* and *S. schinzianum*. **(A)** Types and frequencies of SSRs in the *S. indicum* and *S. schinzianum*. **(B)** Classification and counts of repeat elements in the *S. indicum* and *S. schinzianum* mitogenome.

The numbers of SSRs detected in the mitochondrial genomes of *S. indicum* and *S. schinzianum* were 210 and 184, respectively. Among these, tetranucleotide SSRs were the most abundant, accounting for 44.29% and 50% of the total SSRs in the two species. Dinucleotide SSRs showed the greatest variation between the two genomes, with only 2 (0.95%) detected in *S. indicum* compared to 33 (17.93%) in *S. schinzianum*. In addition, one hexanucleotide SSR was identified in each genome, although their motif compositions differed (**Figure 2A**; **Supplementary Table 2**). A total of 15 and 10 tandem repeats were detected in *S. indicum* and *S. schinzianum*, respectively. The lengths of tandem repeats ranged from 13 to 34 bp in *S. indicum* and from 11 to 22 bp in *S. schinzianum* (**Figure 2B**; **Supplementary Table 3**). Furthermore, 1,787 and 715 dispersed repeats (≥30 bp) were identified in *S. indicum* and *S. schinzianum*, respectively, representing approximately a 2.5-fold difference between the two species. Only one palindromic repeat was detected in *S. schinzianum*, whereas no complementary repeats were identified in either genome (**Figure 2B**; **Supplementary Table 4**).

**Figure 3:**
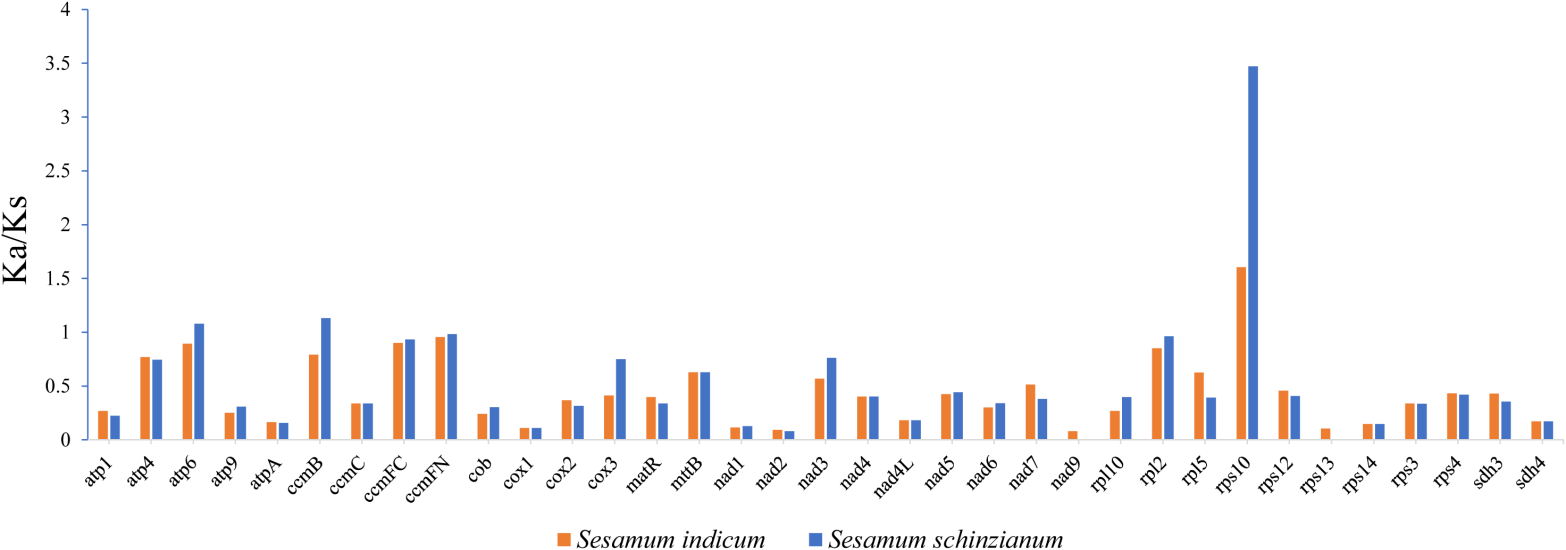
Ka/Ks ratio calculated for the 35 shared genes from the mitochondrial genome of *Sesamum* species.

Ka/Ks ratios were calculated for mitochondrial PCGs between the newly assembled *S. indicum* and *S. schinzianum* mitogenomes (**Figure 3**; **Supplementary Table 5**). Overall, most mitochondrial PCGs showed Ka/Ks values lower than 1, indicating that these genes were generally subject to purifying selection. A small number of genes showed relatively elevated Ka/Ks values, including *atp*6, *ccmB*, and *rps*10, suggesting possible lineage-specific sequence divergence or relaxed selective constraints. In contrast, genes such as *cox*1, *nad*2, *nad*9, *rps*13 and *rps*14 showed relatively low Ka/Ks values, indicating high sequence conservation. These results suggest that purifying selection predominates in mitochondrial PCGs between *S. indicum* and *S. schinzianum*, although a few genes may have experienced relatively accelerated sequence evolution.

### 3.3 Codon usage and RNA editing prediction

**Figure 4:**
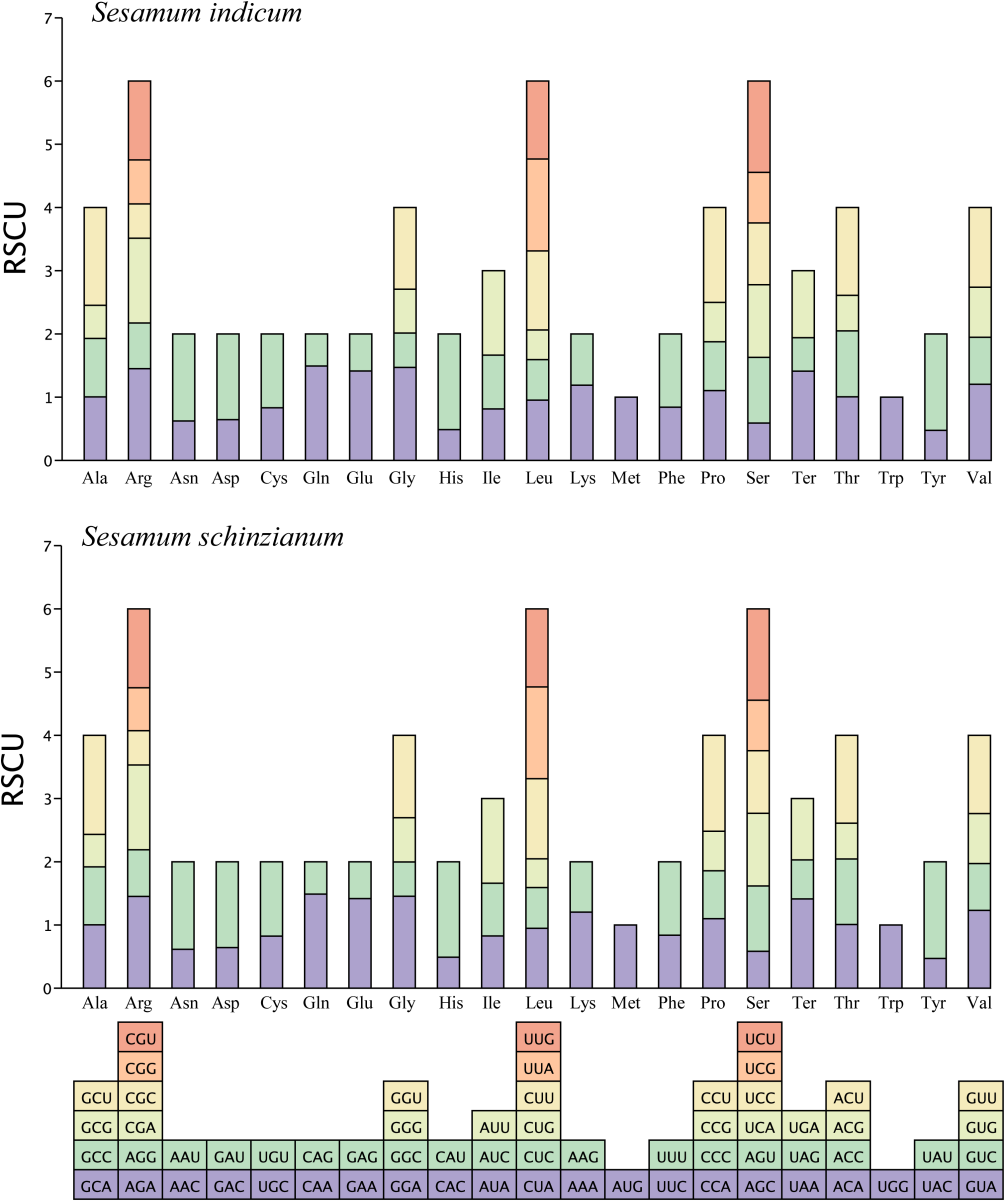
Relative synonymous codon usage (RSCU) of protein-coding genes (PCGs) in the mitochondrial genomes of *S. indicum* and *S. schinzianum*. Codons encoding the same amino acid are distinguished by different colors.

The mitochondrial genome PCGs of *S. indicum* and *S. schinzianum* encode 20 amino acids (**Figure 4**). Nearly half of the codons exhibited usage bias, with relative synonymous codon usage (RSCU) values greater than 1. Analysis of stop codons indicated that UAA, UGA, and UAG are present in both genomes, with UAA being the preferred termination signal (RSCU = 1.41). Additionally, the codons GCA (Ala), AUG (Met), and UGG (Trp) showed RSCU values of 1 in both genomes, suggesting no usage bias (**Supplementary Table 6**). Among all codons, GCU (Ala) was the most frequently used, with RSCU values of 1.55 and 1.57 in *S. indicum* and *S. schinzianum*, respectively, followed by UAU (Tyr), both with RSCU values of 1.53. The least frequently used codons were CUG (Leu) and UAC (Tyr), with RSCU values not exceeding 0.47 (**Supplementary Table 6**).

**Figure 5:**
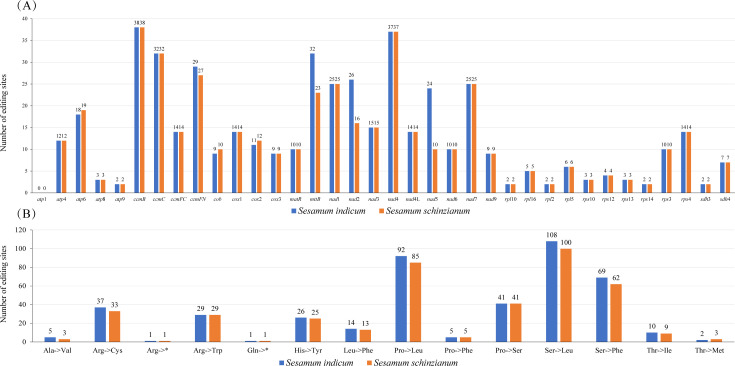
Distribution of RNA editing sites and editing-induced amino acid changes in the mitochondrial genomes of *S. indicum* and *S. schinzianum*. **(A)** Distribution of RNA editing sites in mitochondrial protein-coding genes (PCGs). **(B)** Frequency of amino acid changes induced by RNA editing.

Across the 36 mitochondrial PCGs, 478 predicted RNA editing sites were detected in *S. indicum*, whereas 455 were identified in *S. schinzianum* (**Supplementary Table 7**). Among these genes, the largest numbers of editing sites were found in *ccmB* (38 sites) and *nad*4 (37 sites), while *atp*9, *rp*l2, *rpl*10, *rps*4, and *sdh*3 showed the lowest levels of editing, with only two sites each (**Figure 5A**). No candidate editing site was detected in *atp*1 in either species. Most predicted editing events caused amino acid changes from Serine (Ser) and Proline (Pro) to Leucine (Leu), whereas the rarest changes involved conversions of Arginine (Arg) and Glutamine (Gln) into stop codons (**Figure 5B**).

### 3.4 Intracellular DNA transfer from chloroplast to mitochondrial genomes

**Figure 6:**
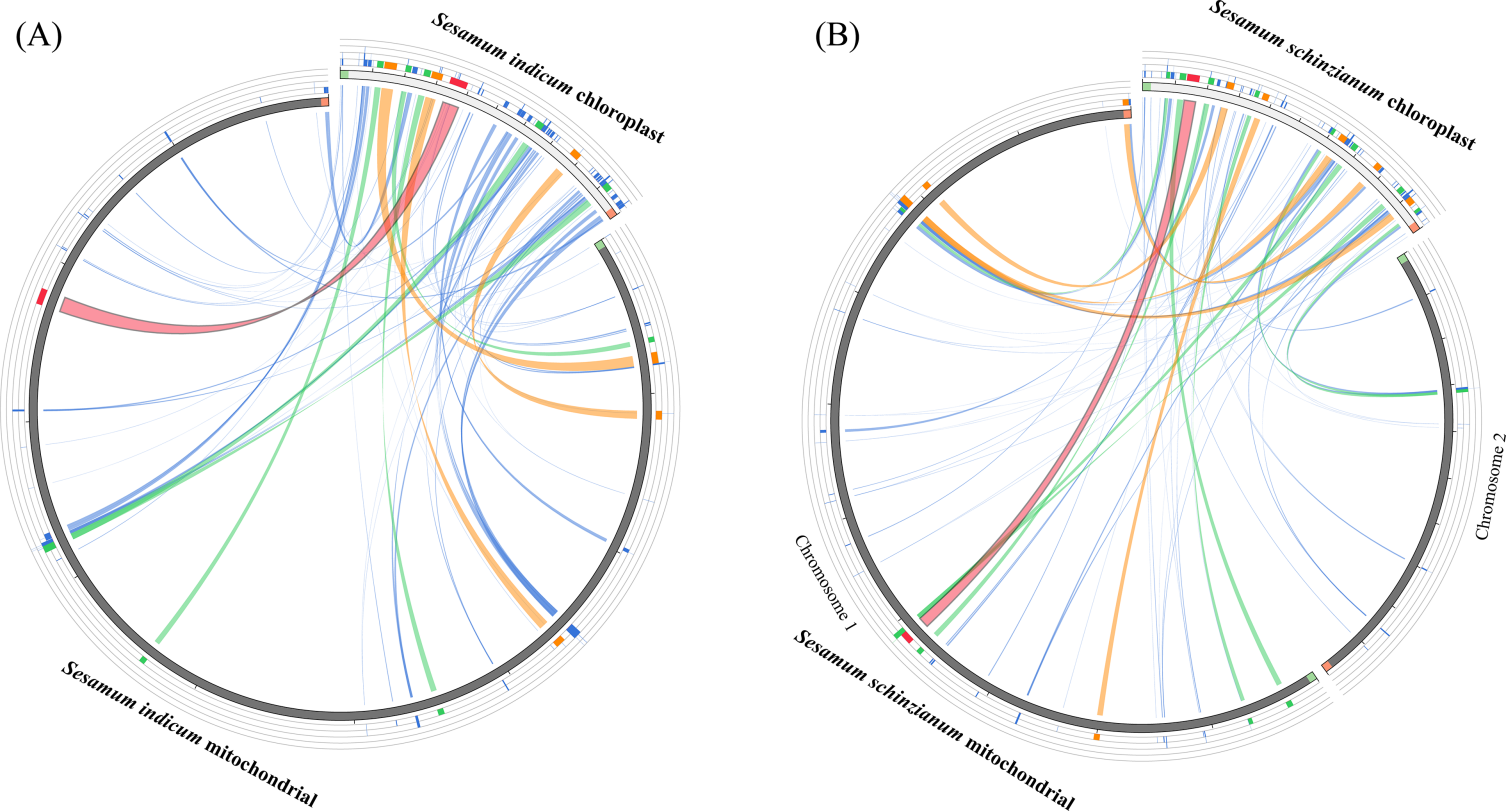
Homology between mitochondrial and chloroplast genomes of *S. indicum* **(A)** and *S. schinzianum* **(B)**. The circular tracks represent mitochondrial and chloroplast genome sequences, and the internal ribbons indicate local alignments identified by BLASTN. Ribbon colors represent quartiles of BLAST bit scores relative to the maximum score: blue indicates the lowest-scoring quartile, followed by green, orange, and red, with red representing the highest-scoring quartile.

A total of 70 and 68 homologous fragments shared with the chloroplast genome were identified in the mitochondrial genomes of *S. indicum* and *S. schinzianum*, respectively (**Figure 6**; **Supplementary Table 8**). These transferred fragments were defined as mitochondrial plastid DNA sequences (MTPTs). In *S. indicum*, the lengths of MTPTs ranged from 40 to 8,121 bp, with a total length of 71,379 bp, accounting for 8.50% of the mitochondrial genome. In *S. schinzianum*, MTPTs ranged from 29 to 5,699 bp, with a total length of 57,623 bp, representing 6.96% of the mitogenome.

Annotation of these homologous regions identified 33 complete genes (excluding duplicates) in *S. indicum*, including 18 PCGs, 14 tRNA genes, and one rRNA gene (**Supplementary Table 8**). In *S. schinzianum*, 26 complete genes (excluding duplicates) were detected, comprising 11 PCGs, 14 tRNA genes, and one rRNA gene (**Supplementary Table 8**). Notably, *trnV-UAC*, *atpB*, *rbcL*, and *ycf*3 were uniquely identified in *S. indicum*, whereas *trnA-UGC*, *psaA*, and *trnP-UGG* were specific to *S. schinzianum*, highlighting differences in MTPT-derived gene content between the two species.

### 3.5 Phylogenetic and collinearity analyses

**Figure 7:**
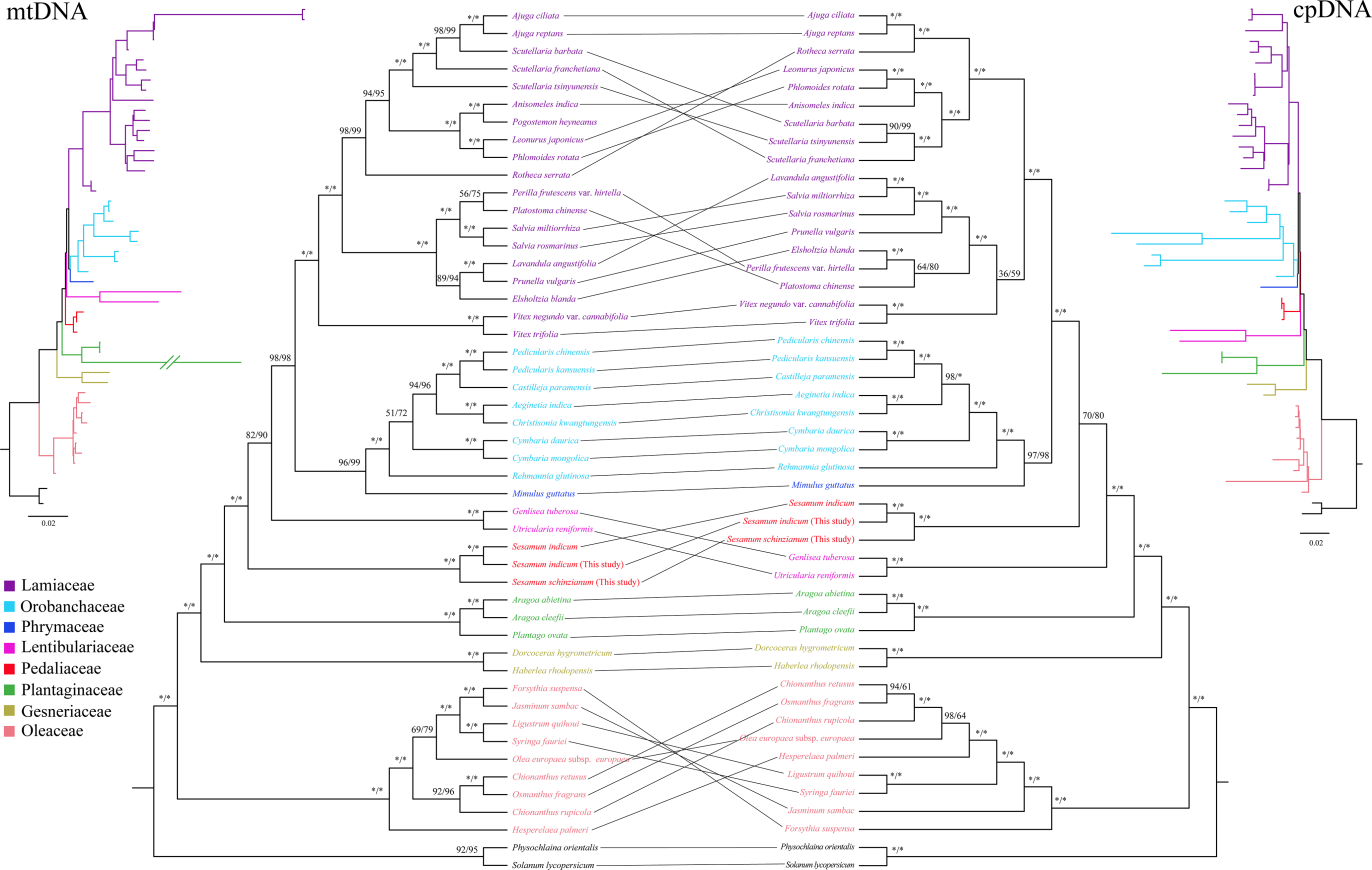
Comparative analysis of maximum likelihood (ML) phylogenetic trees inferred from mitochondrial and plastid protein-coding genes (PCGs) in *Sesamum* and related taxa. Numbers at nodes indicate SH-aLRT / ultrafast bootstrap support values. Asterisks (*) denote support values of 100.

Based on the DNA sequences of 38 conserved mitochondrial PCGs, we inferred a phylogenetic tree for 48 species representing nine families (**Figure 7**). The phylogenetic analysis supported a well-resolved topology within Lamiales. Lamiaceae clustered with the clade comprising Orobanchaceae and Phrymaceae, and this group was sister to Lentibulariaceae. Pedaliaceae (represented by *Sesamum*) formed a sister lineage to this clade, while Plantaginaceae, Gesneriaceae, and Oleaceae diverged successively as more distant lineages. Within Pedaliaceae, the published multipartite *S. indicum* mitogenome (GenBank accession PP444661-PP444682) clustered together with the *S. indicum* mitogenome assembled in this study, and together they formed a well-supported sister clade (SH-aLRT = 100, UFBoot = 100) to *S. schinzianum*. This clade was recovered as the sister lineage to *S. schinzianum* (SH-aLRT = 100, UFBoot = 100). Comparative analysis of the phylogenetic trees constructed from mitochondrial and chloroplast genomes showed that the two datasets were largely congruent at the family level, with only Pedaliaceae and Lentibulariaceae exhibiting conflicting placements between the two trees. However, marked topological incongruence between the mtDNA and cpDNA trees was particularly evident in the internal relationships of Lamiaceae and Oleaceae, where multiple crossing connections were observed among corresponding species. In both trees, *Sesamum indicum* (this study) was placed within Pedaliaceae and clustered closely with the published *S. indicum* and *S. schinzianum*, indicating a stable phylogenetic position of *Sesamum* across the two organellar genomes.

**Figure 8:**
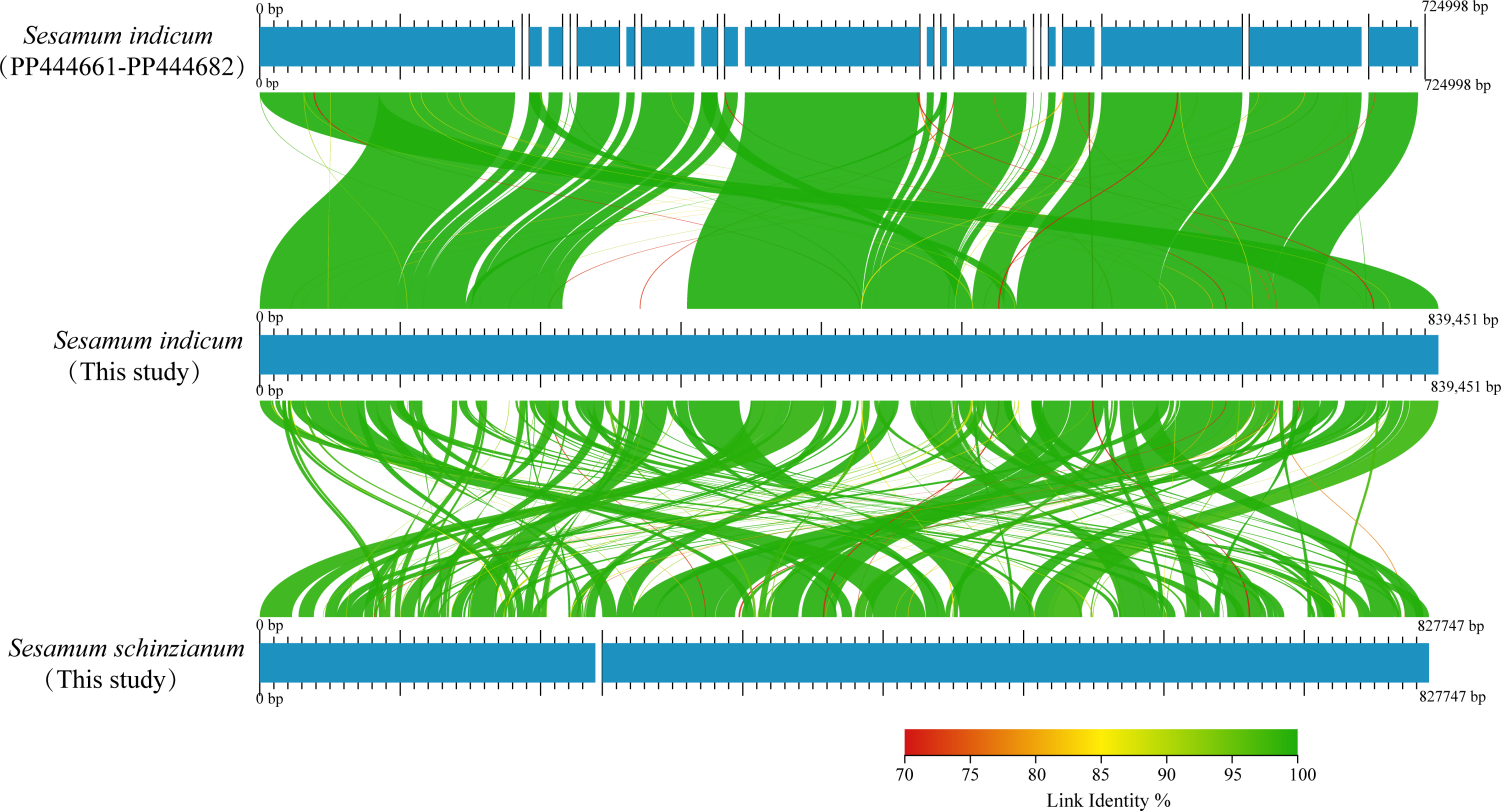
Collinearity analysis of three *Sesamum* species. Arcs colored from red to green represent sequence similarity ranging from 70% to 100%; only alignments longer than 200 bp are shown.

Comparative collinearity analysis of the mitochondrial genomes revealed a high level of synteny between the two *S. indicum* mitogenomes (**Figure 8**). However, an approximately 10 kb fragment identified in the *S. indicum* mitogenome assembled in this study did not correspond to any syntenic region in the previously published *S. indicum* mitochondrial genome. This discrepancy may reflect incomplete assembly or missing sequences in the earlier mitogenome. In contrast, the mitochondrial genomes of *S. indicum* and *S. schinzianum* also shared extensive homologous regions, indicating an overall conserved genomic composition. However, the syntenic blocks between the two species were more fragmented and displayed a more complex arrangement, reflecting substantial structural differences between their mitochondrial genomes. These differences are likely attributable to genome rearrangements.

## 4 Discussion

### 4.1 Genomic features of the *S. indicum* and *S. schinzianum* mitogenome

Angiosperm mitochondrial genomes exhibit remarkable size variation, ranging from approximately 66 kb to 11.3 Mb, and typically contain between 19 and 64 genes (Sloan et al., 2012; Skippington et al., 2015). In our study, the mitogenome of *S. indicum* was resolved as one circular chromosome of 839,451 bp. By contrast, the mitogenome of *S. schinzianum* was assembled into two circular chromosomes measuring 588,761 bp and 238,986 bp, respectively, giving a total length of 827,747 bp. A previously published *S. indicum* mitogenome (GenBank accession PP444661-PP444682) was reported as a multipartite structure comprising 22 circular molecules with a total length of 724,998 bp (Wang et al., 2024), which is smaller in size but structurally more complex than the assembly obtained in this study. In the closely related family Lentibulariaceae, the mitochondrial genomes of *Genlisea tuberosa* (729,765 bp) and *Utricularia reniformis* (857,234 bp) are both represented as single circular molecules (Silva et al., 2017; Matos et al., 2022), showing genome sizes comparable to those of *Sesamum* in Pedaliaceae. In contrast, species from another related family, Plantaginaceae, such as *Aragoa abietina* and *A. cleefii*, possess much smaller mitogenomes of approximately 365 kb and 366 kb, respectively (Mower et al., 2021). Despite substantial variation in genome size and structural organization, the GC content of these mitochondrial genomes remains relatively conserved. While GC content appears to be stable during angiosperm evolution, the diverse structural configurations of mitochondrial genomes may reflect adaptive evolutionary strategies.

In the mitochondrial genome of plants, there are usually a set of 24 core genes that are conserved (Adams et al., 2002). Comparative analysis of the three *Sesamum* mitogenomes revealed that their core protein-coding genes are highly conserved, with all three genomes containing an identical core gene set, indicating strong functional stability within the genus. In contrast, variation was observed in the non-core gene fraction, primarily involving differences in the composition and copy number of tRNA genes. For example, compared with the previously published *S. indicum* mitogenome (Wang et al., 2024), the *S. indicum* genome assembled in this study exhibited increased copy numbers of several tRNA genes, including *trnL-CAA*, *trnS-GGA*, *trnS-UGA*, *trnT-UGU*, *trnY-GUA*, and *trnfM-CAU*. In addition, the *S. schinzianum* mitogenome uniquely contained the *trnA-UGC* gene, which was absent in *S. indicum*. Although the rRNA gene repertoire was conserved across the three genomes, variation in copy number was observed for *rrn*26. These differences are mainly reflected in tRNA gene copy number variation and the gain or loss of individual genes, which are common features in plant mitochondrial genomes and may be associated with repeat-mediated genome rearrangements, intracellular DNA transfer, or gene loss during evolution.

### 4.2 Repeat sequences and homologous recombination in the *S. indicum* and *S. schinzianum* mitogenome

Repetitive sequences are ubiquitous and highly polymorphic components of plant mitochondrial genomes, playing important roles in genome rearrangement, inversion, insertion, and deletion. Simple sequence repeats (SSRs), typically composed of short motifs ranging from 1 to 6 bp, are particularly valuable due to their high polymorphism, relative abundance, codominant inheritance, and broad genomic distribution (Schlötterer, 2004). In this study, the numbers of SSRs detected in the mitogenomes of *S. indicum* and *S. schinzianum* reached 210 and 184, respectively, suggesting that these loci may serve as informative markers for species discrimination and genetic diversity assessment in *Sesamum*. Notably, tetranucleotide SSRs were highly abundant in both species, accounting for 44.29% and 50% of total SSRs, respectively, which is higher than commonly observed in most plant mitogenomes. This pattern suggests a lineage-specific accumulation of repetitive elements in *Sesamum*. Long repeat sequences, including tandem, palindromic, forward, reverse, and complementary types, are widely distributed across genomes and are closely associated with gene regulation, maintenance of genome stability, and evolutionary processes (Yang et al., 2013; Hannan, 2018).

Synteny analysis showed strong collinearity between the *S. indicum* mitogenomes assembled in this study and the previously published multipartite *S. indicum* mitogenome, suggesting that the major genomic content is largely conserved within the species. However, an approximately 10 kb fragment identified in the *S. indicum* genome assembled in this study lacked a corresponding syntenic region in the previously published genome, which may partly explain the difference in total genome size between the two assemblies. In addition, the single circular representation of the *S. indicum* mitogenome obtained in this study contrasts with the previously reported multipartite structure consisting of 22 circular molecules (Wang et al., 2024). This contrast may reflect differences in assembly strategy, assembly completeness, or assembly- and synteny-based structural differences, rather than necessarily indicating distinct *in vivo* mitochondrial conformations. In comparison between *S. indicum* and *S. schinzianum*, extensive homologous regions were retained, suggesting conserved mitochondrial genomic content within *Sesamum*. Nevertheless, the increased number and more complex arrangement of syntenic blocks suggest possible interspecific differences in mitochondrial genome organization. These patterns may be associated with repeat-mediated genome rearrangements, DNA insertion and DNA loss events (Palmer et al., 2000), although further breakpoint-level validation or additional long-read evidence would be needed to confirm the underlying mechanisms. Overall, the synteny-based comparison provides useful evidence for conserved genomic content and potential structural dynamics in *Sesamum* mitochondrial genomes, offering insights into mitochondrial genome organization and evolutionary patterns within the genus.

### 4.3 Codon usage and RNA editing in the *S. indicum* and *S. schinzianum* mitogenome

RSCU is an indicator used to quantify the bias in the usage of synonymous codons within a gene (Plotkin and Kudla, 2011). Codon usage bias was observed in the mitochondrial genomes of *S. indicum* and *S. schinzianum*, with nearly half of the codons showing RSCU values greater than 1. The preference for UAA as the stop codon and the frequent use of A/U-ending codons (e.g., GCU and UAU) are consistent with patterns commonly reported in plant mitochondrial genomes. These results suggest that codon usage in *Sesamum* mitogenomes is shaped by underlying nucleotide composition and evolutionary constraints.

In plant mitochondria, RNA editing is commonly observed as a post-transcriptional event (Edera et al., 2018), and it may partially compensate for DNA mutations through changes introduced at the RNA level (Takenaka et al., 2013). In *S. indicum* and *S. schinzianum*, 478 and 455 putative RNA editing sites were predicted across 36 PCGs, respectively, and all predicted sites corresponded to typical C-to-U conversions, consistent with the predominant editing pattern in plant organelles (Small et al., 2020). The distribution of predicted editing sites was uneven, with *ccmB* and *nad*4 containing the most sites, whereas several genes (e.g., *atp*9, *rpl*2, *rpl*10, *rps*4, and *sdh*3) had only a few, and no editing was detected in *atp*1, indicating gene-specific variation in predicted editing-site distribution. Most editing events resulted in amino acid changes from Ser and Pro to Leu, while conversions to stop codons were rare, which may be related to mitochondrial protein sequence conservation.

### 4.4 Chloroplast-derived sequences and selection pressure in the mitogenomes of *S. indicum* and *S. schinzianum*

Intracellular gene transfer between organellar genomes plays a crucial role in plant evolution (Sloan and Wu, 2014). In most plants, mitochondrial plastid sequences (MTPTs) account for approximately 1–10% of the mitochondrial genome (Mower et al., 2012). In this study, MTPTs constituted 8.50% of the *S. indicum* mitogenome and 6.96% of the *S. schinzianum* mitogenome, indicating that chloroplast-to-mitochondria DNA transfer in *Sesamum* is consistent with the typical range observed in other angiosperms. Both species’ MTPTs include multiple tRNA, rRNA, and protein-coding genes, with several tRNA genes being transferred, which is a common phenomenon in plants (Bergthorsson et al., 2003). Notably, some MTPT genes are species-specific, including *trnV-UAC*, *atpB*, *rbcL* and *ycf*3 in *S. indicum*, as well as *trnA-UGC*, *psaA* and *trnP-UGG* in *S. schinzianum*, highlighting differences in chloroplast-derived gene content between the two species. These observations suggest that, while the overall level of plastid DNA integration is conserved, the composition of transferred genes varies, potentially contributing to mitochondrial genome structural variation and the diversification of *Sesamum* species.

The Ka/Ks analysis indicated that most mitochondrial PCGs in *S. indicum* and *S. schinzianum* had values below 1, suggesting that purifying selection is the predominant evolutionary force acting on mitochondrial PCGs in these two *Sesamum* species. This pattern is consistent with the generally conserved nature of plant mitochondrial coding sequences, which are often functionally constrained despite extensive variation in mitochondrial genome structure. Nevertheless, a small number of genes, including *atp*6, *ccmB*, and *rps*10, showed relatively elevated Ka/Ks values in the interspecific comparison. In particular, the elevated Ka/Ks value of *rps*10 suggests that this gene may have experienced relatively accelerated sequence divergence or relaxed selective constraint between *S. indicum* and *S. schinzianum*. However, this signal should be interpreted cautiously because high Ka/Ks estimates in mitochondrial genes may also be influenced by low synonymous substitution rates, alignment uncertainty, gene length, or annotation differences (Rocha et al., 2006; Schneider et al., 2009; Jordan and Goldman, 2012; Redelings, 2014). Therefore, these genes should be considered candidate loci for further investigation rather than definitive evidence of positive selection. Overall, these results suggest that purifying selection predominates in *S. indicum* and *S. schinzianum* mitogenomes, while a small subset of genes may have experienced distinct evolutionary trajectories (Palmer et al., 2000).

### 4.5 Phylogeny of the *S. indicum* and *S. schinzianum* mitogenome

Plant mitochondrial DNA is generally conserved and evolves at a relatively slow nucleotide substitution rate, making it informative for phylogenetic inference (Drouin et al., 2008). At the same time, plant mtDNA is often larger and structurally more dynamic than chloroplast DNA, which may provide additional phylogenetic information (Evans et al., 2019). In this study, phylogenetic analysis based on conserved mitochondrial protein-coding genes recovered a largely resolved topology within Lamiales. Within Pedaliaceae, the published multipartite *S. indicum* mitogenome clustered with the *S. indicum* mitogenome assembled in this study, and together they formed a well-supported sister clade to *S. schinzianum*, supporting a close relationship among these taxa. Comparisons between mitochondrial and plastid phylogenies showed overall congruence at the family level, but topological differences were observed in several deeper nodes, particularly in the placement of Pedaliaceae and Lentibulariaceae and in the internal relationships of Lamiaceae and Oleaceae. Such differences are not unexpected, because some deep relationships within Lamiales remain difficult to resolve consistently across datasets (Schäferhoff et al., 2010; Refulio-Rodriguez and Olmstead, 2014). These differences may be related to the distinct evolutionary dynamics and phylogenetic signals of mitochondrial and plastid genomes; in addition, biological processes such as hybridization, introgression, organelle capture, and incomplete lineage sorting cannot be excluded, although they cannot be directly inferred from the present organellar datasets (Palmer et al., 2000; Camus et al., 2022; Tyszka et al., 2023; Wanke and Wicke, 2023). Therefore, the conflict observed here should be interpreted cautiously and further assessed using expanded taxon sampling and nuclear genomic data.

## 5 Conclusion

In this study, the mitochondrial genomes of *S. indicum* and *S. schinzianum* were assembled and comparatively analyzed. Both genomes showed conserved gene content but differed in structural organization, repeat composition, and MTPT-derived sequences. Phylogenetic analysis provided additional insight into the placement of Pedaliaceae and confirmed the close relationship between the two *Sesamum* species. Moreover, comparison between mitochondrial- and chloroplast-based phylogenies showed overall congruence at the family level but revealed topological incongruence in several deeper nodes, suggesting that the two organellar genomes retain partially distinct evolutionary signals. Overall, these findings highlight the dynamic structural evolution and conserved functional features of *Sesamum* mitogenomes, and provide valuable resources for future evolutionary, phylogenetic, and comparative genomic studies.

## Data Availability

The mitochondrial genome of *S. indicum* has been deposited in GenBank under accession number PZ173270, while the mitochondrial genomes of *S. schinzianum* are available under accession numbers PZ179903–PZ179904. The plastid genomes of *S. indicum* and *S. schinzianum* have been deposited in GenBank under accession numbers PZ173271 and PZ173272, respectively.
