# Supplementary figures and images for: Comparative mitochondrial genomics of cultivated Sesamum indicum and its wild relative Sesamum schinzianum provides insights into structural features and organellar evolution
Source: Front Plant Sci. 2026 Jun 10;17:1858359. doi: 10.3389/fpls.2026.1858359 (PMC13290937; doi:10.3389/fpls.2026.1858359)

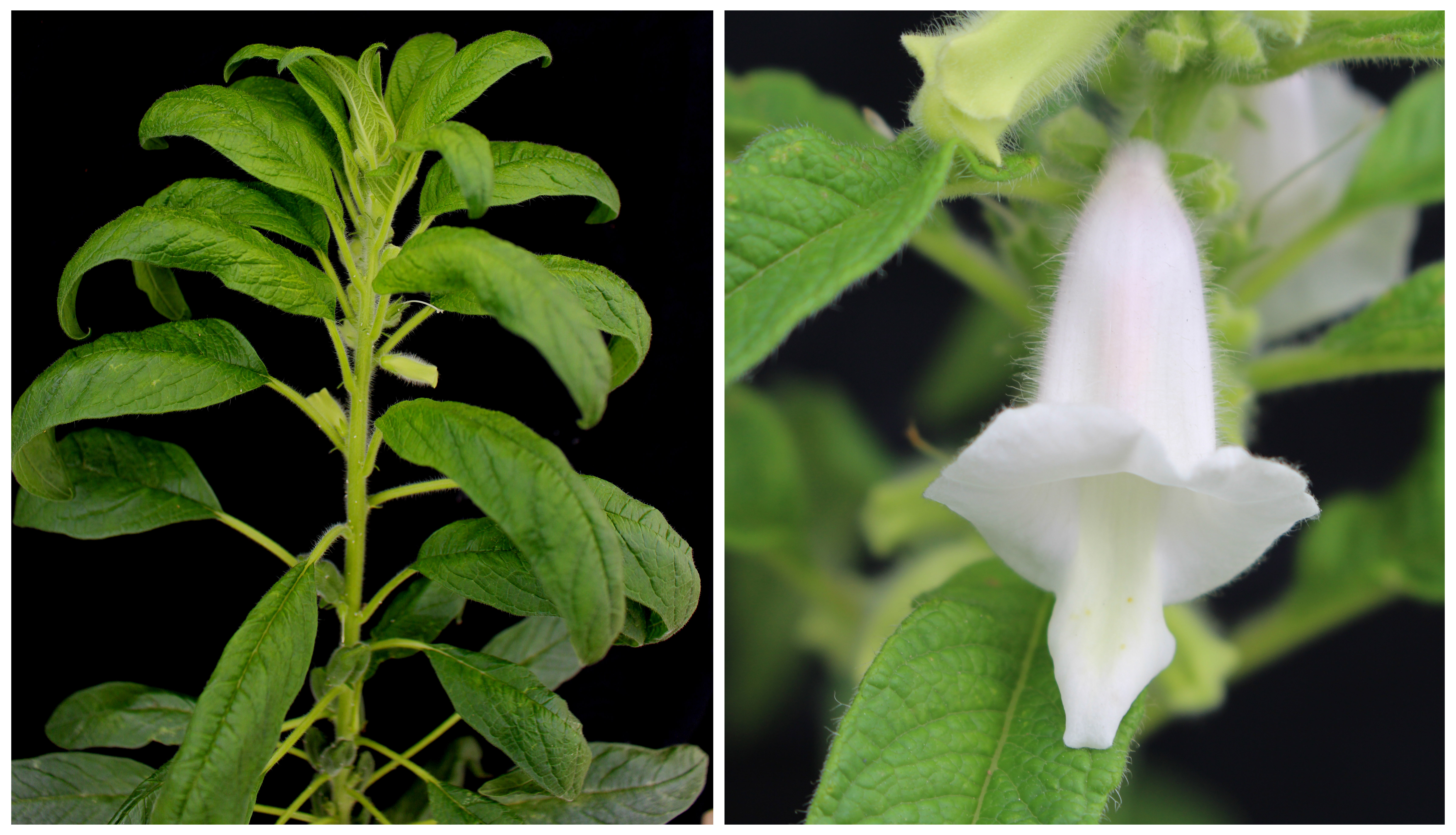

Supplement: Supplementary file 1 [file Image1.jpeg]

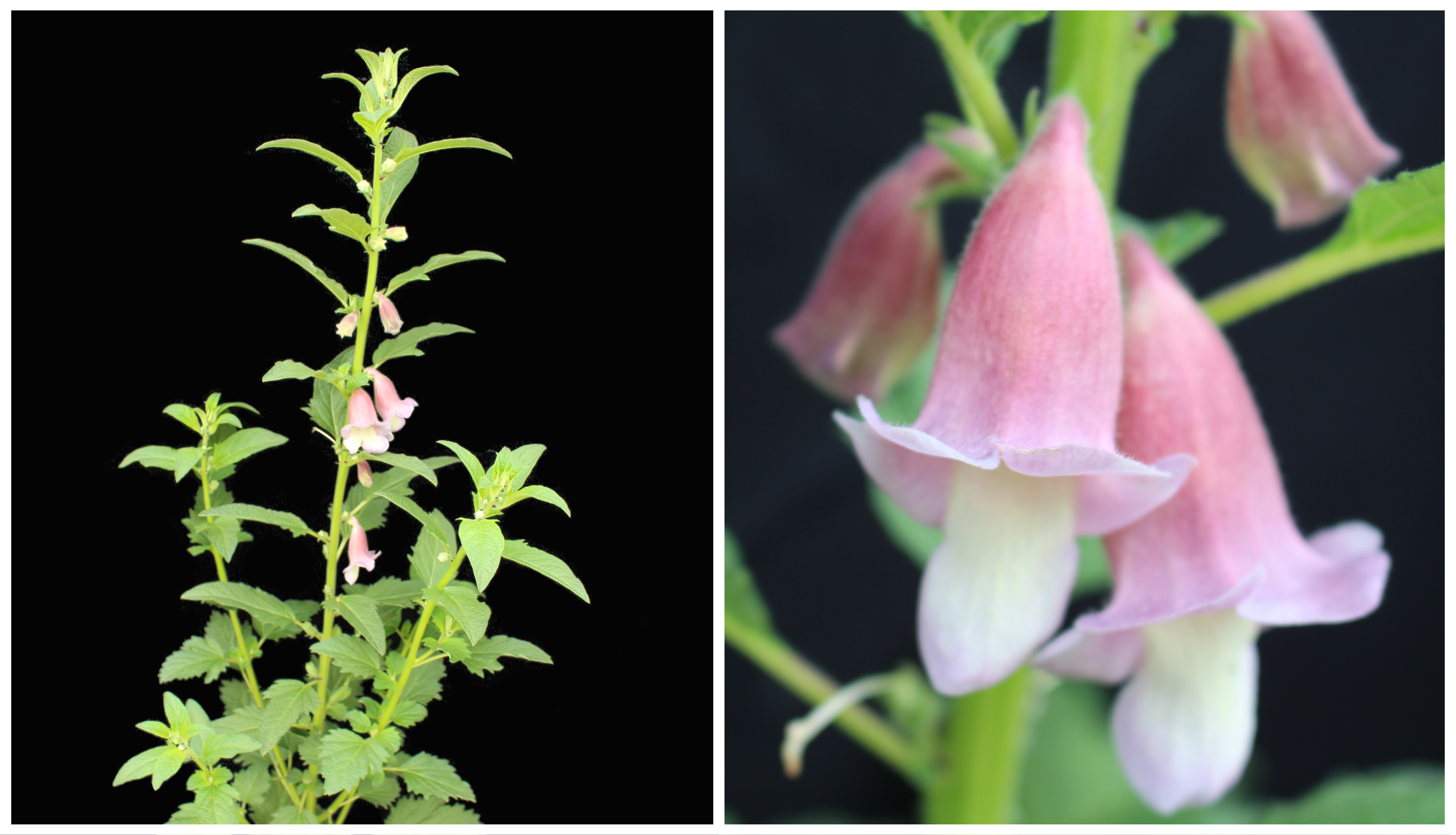

Supplement: Supplementary file 2 [file Image2.jpeg]
